# Incorporating background frequency improves entropy-based residue conservation measures

**DOI:** 10.1186/1471-2105-7-385

**Published:** 2006-08-17

**Authors:** Kai Wang, Ram Samudrala

**Affiliations:** 1 Computational Genomics Group, Department of Microbiology, University of Washington, USA

## Abstract

**Background:**

Several entropy-based methods have been developed for scoring sequence conservation in protein multiple sequence alignments. High scoring amino acid positions may correlate with structurally or functionally important residues. However, amino acid background frequencies are usually not taken into account in these entropy-based scoring schemes.

**Results:**

We demonstrate that using a relative entropy measure that incorporates amino acid background frequency results in improved performance in identifying functional sites from protein multiple sequence alignments.

**Conclusion:**

Our results suggest that the application of appropriate background frequency information may lead to more biologically relevant results in many areas of bioinformatics.

## Background

Protein multiple sequence alignments are widely used to infer conservation of amino acid residues within an evolutionarily related family [1,2]. Highly conserved residues tend to correlate with structural and/or functional importance, and accurate identification of such important residues aids in experimental characterization of protein function.

One commonly used sequence conservation measure is the entropy score. In its simplest form, the entropy score for each aligned column in a multiple sequence alignments can be expressed as:

Sentropy=H=−∑inaapilog⁡2pi     (1)
 MathType@MTEF@5@5@+=feaafiart1ev1aaatCvAUfKttLearuWrP9MDH5MBPbIqV92AaeXatLxBI9gBaebbnrfifHhDYfgasaacH8akY=wiFfYdH8Gipec8Eeeu0xXdbba9frFj0=OqFfea0dXdd9vqai=hGuQ8kuc9pgc9s8qqaq=dirpe0xb9q8qiLsFr0=vr0=vr0dc8meaabaqaciaacaGaaeqabaqabeGadaaakeaacqWGtbWudaWgaaWcbaGaemyzauMaemOBa4MaemiDaqNaemOCaiNaem4Ba8MaemiCaaNaemyEaKhabeaakiabg2da9iabdIeaijabg2da9iabgkHiTmaaqahabaGaemiCaa3aaSbaaSqaaiabdMgaPbqabaaabaGaemyAaKgabaGaemOBa42aaSbaaWqaaiabdggaHjabdggaHbqabaaaniabggHiLdGccyGGSbaBcqGGVbWBcqGGNbWzdaWgaaWcbaGaeGOmaidabeaakiabdchaWnaaBaaaleaacqWGPbqAaeqaaOGaaCzcaiaaxMaadaqadiqaaiabigdaXaGaayjkaiaawMcaaaaa@52B7@

where *n*_*aa *_is the number of residue types in the column representing an alignment position, and *p*_*i *_represents the observed frequency of residue type *i *in the aligned column. Other, more complicated, residue conservation measures derived from the entropy score have been developed and used in identifying functionally important residues [3-8].

In recent years many sophisticated functional site prediction algorithms have been developed (for reviews, see [9,10]). Many of these prediction algorithms implicitly or explicitly analyze the amino acid variations in a given position in multiple alignments. The evolutionary trace method analyzes residue variation patterns within and between protein subfamilies from multiple alignments, and maps important residues to protein structure [11,12]. A further development of this method incorporates entropy information for more accurate ranking of residue importance [13]. Oliveira *et al *devised entropy-variability plots that can be used to identify structurally and functionally important residues [14]. Pei *et al *used the conservation difference between artificial sequence profiles and naturally occurring sequence profiles to detect homology and identify active sites [15]. Soyer *et al *used site-specific evolutionary models for predicting functional sites in proteins [16]. Wang *et al *used linear models to analyze multiple alignments for mutated viral proteins to identify amino acid positions important for drug resistance [17]. Chelliah *et al *identified interaction sites by separating the structural and functional constraints for each position in multiple alignments [18]. Greaves *et al *used geometry-based and sequence profile-based calculation for predicting enzyme active sites [19]. Cheng *et al *introduced a hybrid method incorporating both sequence conservation and structural stability to predict functional sites [20]. The SIFT server automatically constructs multiple alignments for query sequence and then predicts amino acid substitutions that are likely to affect protein function [21]. The ConSurf server identifies protein functional region by surface mapping of phylogenetic information inferred from multiple alignments [22]. The MINER server uses phylogenetic motifs from multiple alignments to identify protein functional site regions [23]. Besides functional site identification, the residue conservation information can also be used to identify residues determining subfamiliy functional specificity [24-28]. The prevalence of these methods suggests that the extraction of conservation information from multiple alignments is important for the correct prediction of functional residues or regions.

## Results and discussion

### Rationale

Here, we argue that entropy scores that do not incorporate background amino acid frequencies are not theoretically optimal for calculating residue conservation. To demonstrate this, we rewrite the entropy score using a uniform amino acid frequency distribution *P*_*u *_(*p*_*u *_= 1/*n*_*aa *_for each residue type in the aligned column):

H=−∑inaapilog⁡2pi=−∑inaapi(log⁡2pi−log⁡2pu)−∑inaapilog⁡2pu=−∑inaapilog⁡2(pi/pu)−log⁡2pu=−D(Pi||Pu)−log⁡2pu     (2)
 MathType@MTEF@5@5@+=feaafiart1ev1aaatCvAUfKttLearuWrP9MDH5MBPbIqV92AaeXatLxBI9gBaebbnrfifHhDYfgasaacH8akY=wiFfYdH8Gipec8Eeeu0xXdbba9frFj0=OqFfea0dXdd9vqai=hGuQ8kuc9pgc9s8qqaq=dirpe0xb9q8qiLsFr0=vr0=vr0dc8meaabaqaciaacaGaaeqabaqabeGadaaakeaafaqaaeabbaaaaeaacqWGibascqGH9aqpcqGHsisldaaeWbqaaiabdchaWnaaBaaaleaacqWGPbqAaeqaaaqaaiabdMgaPbqaaiabd6gaUnaaBaaameaacqWGHbqycqWGHbqyaeqaaaqdcqGHris5aOGagiiBaWMaei4Ba8Maei4zaC2aaSbaaSqaaiabikdaYaqabaGccqWGWbaCdaWgaaWcbaGaemyAaKgabeaaaOqaaiabg2da9iabgkHiTmaaqahabaGaemiCaa3aaSbaaSqaaiabdMgaPbqabaaabaGaemyAaKgabaGaemOBa42aaSbaaWqaaiabdggaHjabdggaHbqabaaaniabggHiLdGccqGGOaakcyGGSbaBcqGGVbWBcqGGNbWzdaWgaaWcbaGaeGOmaidabeaakiabdchaWnaaBaaaleaacqWGPbqAaeqaaOGaeyOeI0IagiiBaWMaei4Ba8Maei4zaC2aaSbaaSqaaiabikdaYaqabaGccqWGWbaCdaWgaaWcbaGaemyDauhabeaakiabcMcaPiabgkHiTmaaqahabaGaemiCaa3aaSbaaSqaaiabdMgaPbqabaGccyGGSbaBcqGGVbWBcqGGNbWzdaWgaaWcbaGaeGOmaidabeaakiabdchaWnaaBaaaleaacqWG1bqDaeqaaaqaaiabdMgaPbqaaiabd6gaUnaaBaaameaacqWGHbqycqWGHbqyaeqaaaqdcqGHris5aaGcbaGaeyypa0JaeyOeI0YaaabCaeaacqWGWbaCdaWgaaWcbaGaemyAaKgabeaaaeaacqWGPbqAaeaacqWGUbGBdaWgaaadbaGaemyyaeMaemyyaegabeaaa0GaeyyeIuoakiGbcYgaSjabc+gaVjabcEgaNnaaBaaaleaacqaIYaGmaeqaaOGaeiikaGIaemiCaa3aaSbaaSqaaiabdMgaPbqabaGccqGGVaWlcqWGWbaCdaWgaaWcbaGaemyDauhabeaakiabcMcaPiabgkHiTiGbcYgaSjabc+gaVjabcEgaNnaaBaaaleaacqaIYaGmaeqaaOGaemiCaa3aaSbaaSqaaiabdwha1bqabaaakeaacqGH9aqpcqGHsislcqWGebarcqGGOaakcqWGqbaudaWgaaWcbaGaemyAaKgabeaakiabcYha8jabcYha8jabdcfaqnaaBaaaleaacqWG1bqDaeqaaOGaeiykaKIaeyOeI0IagiiBaWMaei4Ba8Maei4zaC2aaSbaaSqaaiabikdaYaqabaGccqWGWbaCdaWgaaWcbaGaemyDauhabeaaaaGccaWLjaGaaCzcamaabmGabaGaeGOmaidacaGLOaGaayzkaaaaaa@B4B1@

where D(*P*_*i *_|| *P*_*u*_) is commonly referred to as the relative entropy or Kullback-Leibler divergence. Therefore, the entropy score is numerically identical to the negative relative entropy between the observed amino acid frequency distribution *P*_*i *_and a uniform distribution *P*_*u*_, minus a constant. In statistics, relative entropy arises as the expected logarithm of the likelihood ratio, and it may be used as a measure of the distance between two probability distributions [29]. In the case of residue conservation, the higher deviation from the "background" indicates stronger evolutionary constraint, which suggests that this position may perform an important functional role. However, nature does not sample every amino acid equally when creating proteins. Therefore, the simple uniform distribution *P*_*u *_above is not optimal as a reference distribution to evaluate functional importance.

We propose that a relative entropy measure incorporating the observed background frequency from protein sequence databases would be a better measure to capture the functional importance of amino acid residues. More specifically, we propose to use the formula:

Srelative_entropy=D(Pi||Pib)=∑ipilog⁡2(pi/pib)     (3)
 MathType@MTEF@5@5@+=feaafiart1ev1aaatCvAUfKttLearuWrP9MDH5MBPbIqV92AaeXatLxBI9gBaebbnrfifHhDYfgasaacH8akY=wiFfYdH8Gipec8Eeeu0xXdbba9frFj0=OqFfea0dXdd9vqai=hGuQ8kuc9pgc9s8qqaq=dirpe0xb9q8qiLsFr0=vr0=vr0dc8meaabaqaciaacaGaaeqabaqabeGadaaakeaacqWGtbWudaWgaaWcbaGaemOCaiNaemyzauMaemiBaWMaemyyaeMaemiDaqNaemyAaKMaemODayNaemyzauMaei4xa8LaemyzauMaemOBa4MaemiDaqNaemOCaiNaem4Ba8MaemiCaaNaemyEaKhabeaakiabg2da9iabdseaejabcIcaOiabdcfaqnaaBaaaleaacqWGPbqAaeqaaOGaeiiFaWNaeiiFaWNaemiuaa1aaSbaaSqaaiabdMgaPjabdkgaIbqabaGccqGGPaqkcqGH9aqpdaaeqbqaaiabdchaWnaaBaaaleaacqWGPbqAaeqaaaqaaiabdMgaPbqab0GaeyyeIuoakiGbcYgaSjabc+gaVjabcEgaNnaaBaaaleaacqaIYaGmaeqaaOGaeiikaGIaemiCaa3aaSbaaSqaaiabdMgaPbqabaGccqGGVaWlcqWGWbaCdaWgaaWcbaGaemyAaKMaemOyaigabeaakiabcMcaPiaaxMaacaWLjaWaaeWaceaacqaIZaWmaiaawIcacaGLPaaaaaa@6C17@

where *P*_*ib *_can represent the background amino acid frequencies found in naturally occurring protein sequences, or any other arbitrary set of background frequencies. This measure will increase the scores for aligned columns containing "rare" residues, which are often functionally important. To explain this in a more intuitive way, consider two invariant positions that have only cysteines and only serines, respectively. The position with cysteines is more likely to be functional. The entropy measure will assign the same score to the two positions, but the relative entropy measure assigns a higher score to the invariant cysteine position, since cysteine has a much lower background frequency (~2%) than serine (~7%).

### Comparative analysis of entropy score and relative entropy score

To investigate whether our proposed relative entropy measure (*S*_*relative_entropy*_) is more sensitive than the entropy measure (*S*_*entropy*_) in detecting functional sites, we evaluated the performance of these measures to identify functionally important residues using the Thornton [30] and Lovell datasets [18]. In addition, to investigate how the use of different background frequencies affects the performance of the relative entropy method, we used two sets of frequencies: the general background frequencies observed in nature and the family-specific background frequencies retrieved from the alignments for each query sequence.

**Figure 1:**
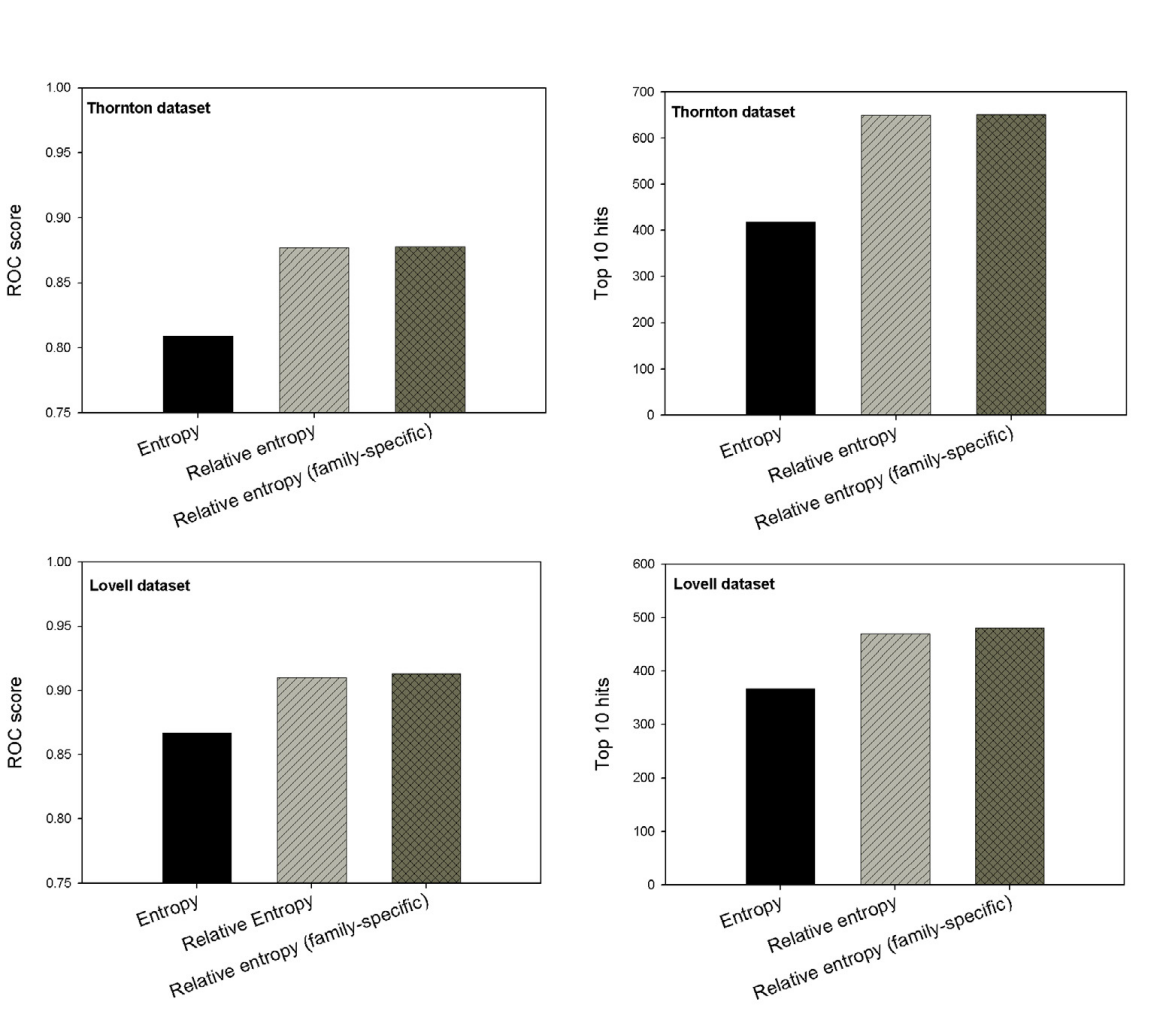
Accuracy of functional site identification by the entropy method, the default relative entropy method and the family-specific relative entropy method on the Thornton and Lovell datasets. The average ROC score and the top 10 hits score are used to evaluate performance. The family-specific relative entropy method has negligible difference in performance compared to the default relative entropy method using general background frequencies. Both relative entropy methods perform better than the entropy method, demonstrating that the incorporation of background frequency information improves functional site identification.

For each protein in the datasets, we built multiple alignments and calculated the entropy or relative entropy scores for each residue and evaluated their performance by two criteria: The first criterion is the ROC score, which measures how the quantitative scores correlate with true functional sites. The second criterion is the "top 10 hits" score, which counts the number of functionally important residues that are in the top 10 highest scoring residues. We found that the relative entropy method significantly outperforms the entropy method under both evaluation criteria for both datasets (Figure 1). In addition, using family-specific background frequencies in the relative entropy method has similar performance to using general background frequencies.

**Figure 2:**
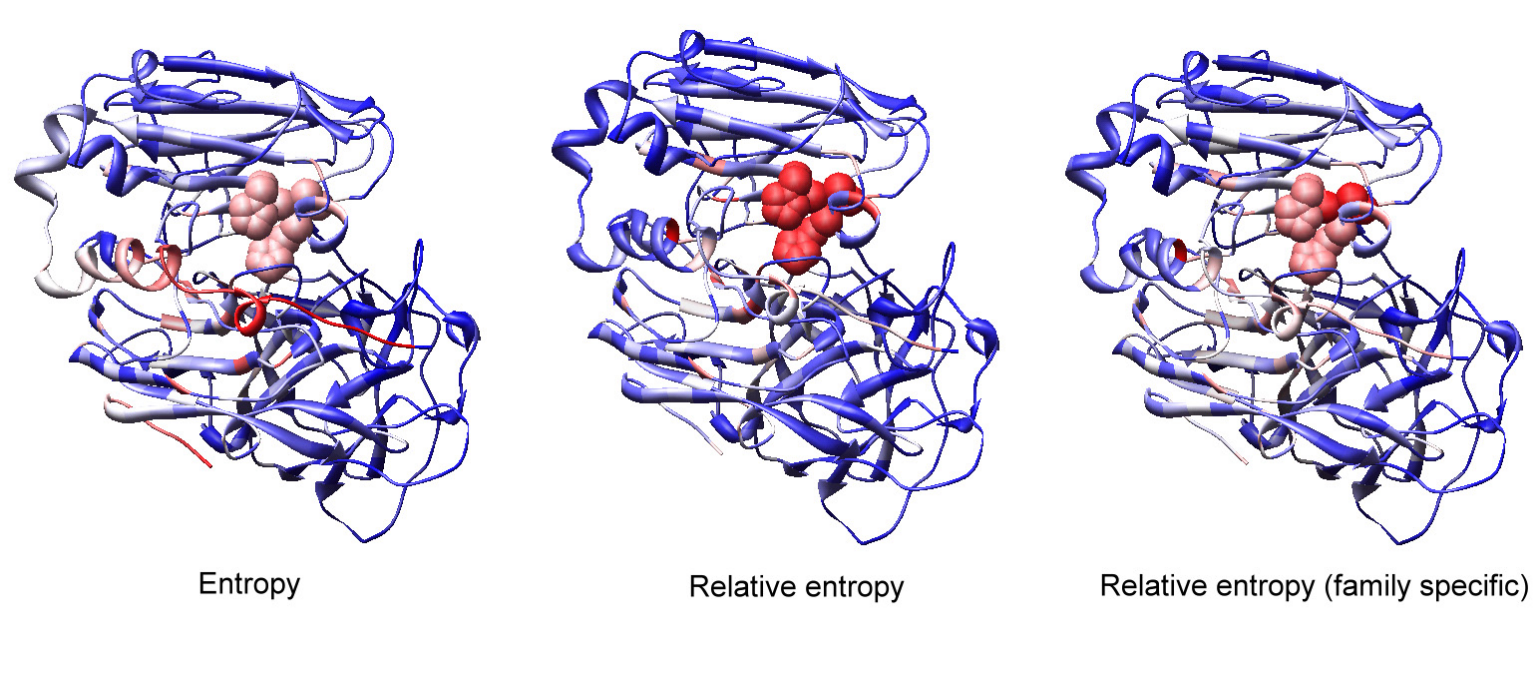
Example comparison of relative entropy and entropy methods. The structure of the example protein, an oxidoreductase (PDB identifier 1a65A), is shown in ribbon representation with the functional active sites (His-Cys-His) represented as spheres. For each method evaluated, each residue in the structure is colored by its predicted functional importance score, with the color changing from red to white to blue as the score decreases. The entropy method incorrectly assigns the highest scores to residues in the C-terminal region, but the two relative entropy methods correctly assign the highest scores to the active sites.

To further investigate why the relative entropy methods perform better than the entropy method, we selected an example from the Thornton dataset where both the relative entropy methods accurately identify the active sites in their "top 10 hits", but where the entropy method fails to do so. This example protein (PDB identifier 1a65A) is an oxidoreductase, and contains three consecutive active sites (His-Cys-His). We plotted the structures as ribbon representation and colored the residues by their scores from each of the three methods (red color indicating high scoring positions) (Figure 2). We found that the entropy method incorrectly assigns the highest scores to the region near the C-terminal of the protein (Asp-Asp-Leu-Pro-Pro-Glu-Ala-Thr-Ser-Ile-Gln-Thr-Val) and not for the residues in the active site (His-Cys-His, depicted as spheres in figure). The frequencies of these amino acids in nature are approximately 5%, 9%, 5%, 6%, 8%, 6%, 7%, 5%, 4% and 6% for Asp, Leu, Pro, Glu, Ala, Thr, Ser, Ile, Gln and Val, respectively, but only 2% and 2% for His and Cys, respectively. Therefore, by applying the relative entropy measure, we down-weighted the C-terminal region and predicted higher scores for the active sites. For the family-specific relative entropy method, the family-specific frequencies are 4% for His and 1% for Cys, so the position with the Cys has a higher score (more intense red color) than the neighboring His, but all three sites are still among the top 10 hits. Our analysis indicates that taking into account background frequencies boosts the scores for positions containing rare amino acids and results in improved performance for identification of functionally important positions.

### Comparative analysis of entropy scores using more accurate frequency estimates

We further investigated whether entropy-based methods could benefit from incorporating more accurate amino acid frequency information. We compiled a HMM model from multiple alignments for each query sequence, and calculated the positional entropy or relative entropy (using the general background frequencies) for each aligned column, similar to a previous study [24]. There are two main advantages of using HMM models: (1) sequences are weighted so that the effects of uneven or biased database sampling are reduced and (2) frequencies of unobserved amino acids can be estimated through the use of Dirichlet mixtures [31].

**Figure 3:**
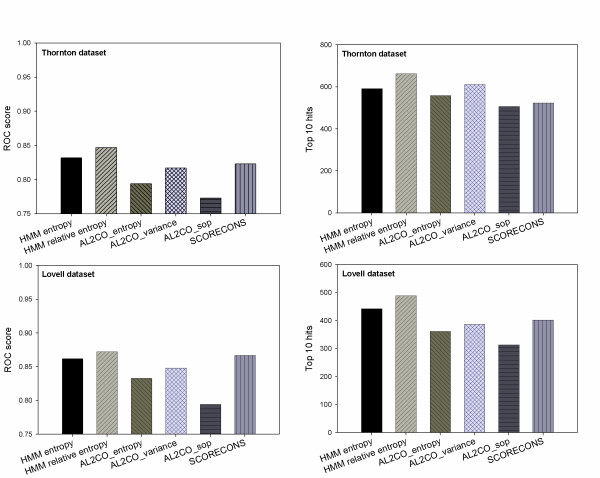
Accuracy of functional site identification by the HMM-derived entropy method, the HMM-derived relative entropy method, three AL2CO-based methods (AL2CO_entropy, AL2CO_variance and AL2CO_sop) and the SCORECONS method on the Thornton and Lovell datasets. The average ROC score and the top 10 hits score are used to evaluate performance. The HMM-derived relative entropy method has the best performance, demonstrating the importance of using background frequency information, as well as accurate estimates of amino acid frequencies.

We evaluated the performance of the HMM-derived entropy and relative entropy methods, as well as several other residue conservation measures, including three AL2CO-based methods [2] and the SCORECONS method [32]. AL2CO is a program that implements an entropy-based method (AL2CO_entropy), a variance-based method (AL2CO_variance) and a sum-of-pairs method (AL2CO_sop). The AL2CO_entropy method is similar to our entropy method; the AL2CO_variance method uses background frequencies that are estimated from the alignment; and the AL2CO_sop method uses pairwise similarity scores derived from a given amino acid substitution matrix. All three AL2CO-based methods apply the default independent count weighting scheme [33] to weight sequences in alignments. The SCORECONS method generates a composite score that takes into account amino acid frequencies, stereochemical diversity, gap penalties, and sequence weighting. This scoring method has been used as a benchmarking score for protein-DNA interaction site identification [32] and protein functional site identification [20]. We found that when HMM-derived amino acid frequencies are used, the relative entropy method still outperforms the entropy method (Figure 3), and both methods outperform the three AL2CO-based methods and the SCORECONS method. Among the three AL2CO-based methods, the AL2CO_variance method performs the best, which may be partially due to its use of background frequencies. The SCORECONS method, which does not outperform the HMM-derived entropy method, was originally developed for scoring conservation in protein-DNA interaction sites and therefore may not be well-suited for predicting functionally important residues in general. In summary, our analysis suggests that using more accurate amino acid frequency estimates, together with using appropriate background frequencies, results in improved functional site prediction from multiple alignments.

Improvements for functional site prediction can occur by increasing the sophistication of the measures considered: The relative entropy method only scores individual positions without considering neighboring residues. A method that analyzes context (neighboring residues) may improve performance. The multiple alignments generated by the PSI-BLAST program may not be optimal, and more accurate multiple alignments (such as those generated by the HMM method) may improve performance. In addition, an appropriate treatment of gaps and sequence weights (for example, a family specific treatment), consideration of phylogenetic relationships among sequences, and analysis of local structural information when available, is also likely to improve performance. We believe that a hybrid method that incorporates the relative entropy method and all the above improvements will have significantly better performance for functional site prediction.

## Conclusion

In conclusion, the use of background frequency information significantly improves entropy-based functional site prediction. This principle has been advocated before (such as in [34]), but its use has been very limited: For example, sequence logo is widely used to visually display conserved nucleotide or amino acid sites in sequence motifs; however, many logo generation programs [35-38] are unable to accept user-supplied background frequencies. (Some exceptions include the PICTOGRAM server [39] and the CONSENSUS server [40].) In addition, several programs for editing or annotating multiple alignments exist [41,42], but they are unable to use background frequencies to calculate relative entropy for each aligned residue.

The use of background information is limited in other areas of bioinformatics as well. For example, many programs are available to identify "functional enrichment" for a list of genes from microarray experiment, but only a few of them are able to accept a user-supplied list of "background genes". Dozens of tools are available to identify transcription factor binding sites (TFBS) or other functional motifs in a given sequence, but few of them are able to take into account the background frequency of predicted TFBS or motifs in the corresponding genome (exceptions include [43]). Fold recognition methods are widely used to assign a query sequence to a structural fold, but few considers the relative abundance (or prior probability) of candidate folds in the corresponding proteome. The broader application of appropriate background information in all areas of bioinformatics will lead to more biologically relevant results.

## Methods

### Data sources

We used two datasets consisting of protein functional sites for evaluating different algorithms. The Thornton dataset was compiled manually from primary literature on known protein structures and was shown to be more comprehensive and specific than the SITE annotation in PDB files [30]. The Lovell dataset contains manually compiled protein functional sites, including ligand binding sites and enzyme active sites [18]. The Thornton dataset contains 1,546 enzyme active sites from 508 proteins, and the Lovell dataset contains 1,137 functional sites from 243 proteins. The same versions of the two sets have been used previously for structure-based searching of functional sites [20].

### Criteria for performance evaluation

We used two criteria for evaluating the performance of functional site prediction algorithms. The first criterion is the ROC score, which computes the area under a curve that plots fraction of true positives versus false positives by varying the threshold value of classification. A perfect classification algorithm that puts all the functional sites at the top of the ranked residue list has an ROC score of 1, and a random classification algorithm has an ROC score of 0.5. The second criterion is the "top 10 hits" score, which computes the number of functional sites among the top 10 scoring residues in a given protein. A perfect classification algorithm has a top 10 hits score of *N*_*fun *_or 10 (if *N*_*fun *_is more than 10), while a random classification algorithm has an average top 10 hits score of 10*N_*fun*_/*N *(*N*_*fun*_and *N *denote the number of functional residues and the number of all residues in the given protein, respectively).

### Functional site identification

We evaluated several functional site identification methods that take protein multiple sequence alignments as input for their predictions. For the Thornton and Lovell datasets, we generated multiple sequence alignments by searching each query sequence against the Uniref90 database [44] with the PSI-BLAST program blastpgp in the BLAST program package [45]. We used three iterations (through the "-j 3" option), the "-m 6" display option and all other default parameters for the PSI-BLAST program. The resulting multiple sequence alignments were converted to ClustalW format, and then analyzed by various scoring methods described below.

For the entropy and relative entropy method, we used equation (1) and (3) in the main text to assign a score to each aligned column in the multiple alignments. We treated gaps in the same way as an amino acid, though we found little performance difference when ignoring gaps. Only the residue types that appear in aligned columns were used in the computation of the relative entropy score. The background distribution *P*_*ib *_in equation (3) can be varied to explore the use of different background frequencies. In our benchmarking experiment, we used two different sets of background frequencies: (1) the general background frequencies defined in karlin.c program of the BLAST package [45], and (2) the family-specific background frequencies observed in the multiple alignments for each query sequence.

For the HMM-derived entropy and relative entropy method, we first compiled a HMM model using the hmmbuild program in the HMMER package [46] with default parameters, and then calculated the positional entropy or relative entropy using amino acid frequencies estimated by the HMM model. The general background frequencies are used for the relative entropy computation.

We also used three conservation measures implemented in the AL2CO program [2], including the entropy measure (AL2CO_entropy), the variance measure (AL2CO_variance) and the sum-of-pairs measure (AL2CO_sop). All the three methods use the default "independent count" scheme for weighting sequences [33]. The default parameters for the AL2CO program were used for computation, except that the BLOSUM62 matrix rather than identity matrix was used in the sum-of-pairs method. For the SCORECONS method [32], we used the scorecons program with default parameters.

## Authors' contributions

KW carried out the computational experiments and drafted the manuscript. RS developed the idea, provided intellectual guidance and mentorship. All authors read and approved the final manuscript.
